# From participation to active aging: the roles of landscape ownership and emotional imprint in community green space regeneration

**DOI:** 10.3389/fpubh.2026.1846490

**Published:** 2026-05-19

**Authors:** Weiwei Zhou, Hui-Ling Hu, Qi Cao

**Affiliations:** 1 Department of Environmental Art Design, College of Science and Technology, Ningbo University, Ningbo, China; 2 Institute of Creative Design and Management, National Taipei University of Business, Taoyuan, Taiwan, China; 3 Graduate School of International Studies, Hanyang University, Seoul, Republic of Korea

**Keywords:** active aging, green space regeneration, meaning in life, participation, place attachment, psychological ownership

## Abstract

**Introduction:**

Population aging poses significant challenges for community planning. While prior research has predominantly examined older adults as passive users of green spaces, less is known about how their direct involvement in green space regeneration is translated into active aging behavior. Drawing on place attachment theory, psychological ownership theory, self-determination theory, and meaning-making perspectives, this study proposes a process-based model in which participation fosters two psychological constructs—emotional imprint on place and sense of landscape ownership—that promote active aging behavior, with meaning in life incorporated as a moderator.

**Methods:**

Survey data were collected from 433 older adults aged 60 and above residing in Chinese communities with experience in green space regeneration initiatives. Partial least squares structural equation modeling (PLS-SEM) was used to estimate the measurement and structural models, with bootstrapping (5,000 resamples) for mediation analysis and a product-indicator approach for moderation.

**Results:**

Perceived participation in green space regeneration exerts a significant positive effect on both emotional imprint on place (β = 0.647, *p* < 0.001) and sense of landscape ownership (β = 0.471, *p* < 0.001), and emotional imprint further predicts landscape ownership (β = 0.367, *p* < 0.001). Both constructs significantly predict active aging behavior (β = 0.290 and β = 0.275, both *p* < 0.01) and partially mediate the participation-behavior relationship. Meaning in life exhibits a dual moderating effect: it strengthens the emotional imprint-behavior link (β = 0.265, *p* < 0.001) but attenuates the landscape ownership-behavior link (β = −0.375, *p* < 0.001).

**Discussion:**

The findings reveal a shift from ownership-based motivation to meaning-driven engagement in later life. By identifying psychological pathways linking participation and behavior, this study advances a place-based psychological perspective on active aging and provides practical implications for community planning that prioritize emotional connection and meaningful engagement over infrastructural provision alone.

## 1 Introduction

Population aging is a defining demographic transition of the twenty-first century. According to the World Health Organization (1), the global population aged 60 and over is projected to double from approximately 1 billion in 2020 to 2.1 billion by 2050, with the most rapid increases concentrated in East Asia, South-East Asia, and parts of Latin America, while many European and North American societies are already navigating advanced stages of this transition (47). Population aging is therefore a shared global challenge: although its tempo and policy responses vary across regions, governments worldwide face common pressures to redesign urban environments, social infrastructure, and community life so that longer lives remain active and meaningful. Within this global context, China represents one of the most pronounced cases of accelerated aging and offers a strategically informative setting for examining how spatial and participatory interventions can support active aging. China formally entered an aging society in 2000, and population aging has since accelerated at an unprecedented pace. According to data from the Seventh National Population Census, individuals aged 60 and above reached 264 million, accounting for 18.7% of the total population (48). This proportion is projected to exceed 400 million by 2035, marking the transition into a phase of deep aging. Such a profound demographic shift poses substantial challenges for urban and rural spatial planning, community development, and public health governance. Promoting the wellbeing and quality of life of older adults has therefore become a central concern in contemporary Chinese society.

At the same time, rapid urbanization has generated a considerable number of underutilized or neglected spaces, including leftover urban fragments and abandoned vacant land. These spaces, often overlooked in conventional planning, have increasingly become focal points for urban regeneration initiatives. In response, local governments and communities across China have actively promoted small-scale interventions such as “micro-renewal,” pocket parks, and community gardens. These practices parallel broader international movements—including pocket-park and tactical-urbanism programmes in North America and Europe, community-garden and allotment traditions across the United Kingdom and continental Europe, and participatory green-space regeneration initiatives in East and South-East Asian cities—that together reframe small, underused urban spaces as sites for civic engagement and everyday wellbeing. While these initiatives reshape the physical landscape, they also carry broader implications for participatory governance and civic engagement (2, 3). Despite these developments, current greening practices in many Chinese communities remain largely top-down. Projects are typically initiated and executed by governmental or commercial actors, with residents positioned as passive recipients rather than active participants (4). This limitation is particularly evident among older adults, whose needs, preferences, and experiential connections to public space are often underrepresented (5). Enabling older residents to engage meaningfully in the regeneration of community spaces may not only enhance their sense of belonging, but also create opportunities to foster more active and engaged forms of aging.

A growing body of literature has demonstrated the positive effects of urban green spaces on older adults' physical and psychological health, including increased physical activity, reduced stress, and enhanced social interaction. However, most of these studies conceptualize older adults primarily as users of space, focusing on accessibility or frequency of use as key determinants (6, 7). Such an approach overlooks the role of older adults as active contributors in the making of space, as well as the psychological processes that may emerge through participation. In particular, relatively little attention has been paid to whether and how direct involvement in the design and regeneration of community spaces may foster deeper emotional attachment and behavioral motivation (8).

Building on this gap, the present study proposes that participation in the greening and regeneration of underutilized community spaces may trigger a series of psychological processes among older adults. Specifically, participation may enhance individuals' perceived sense of control and ownership over space (landscape ownership), as well as facilitate emotional projection and memory embedding within the environment (landscape emotional imprint). These psychological transformations, in turn, may translate into more proactive and sustained forms of engagement, conceptualized in this study as active aging behavior.

Moreover, the extent to which these psychological processes translate into behavior may depend on individuals' sense of meaning in life. When older adults perceive their participation as meaningful—such as contributing to the community, leaving a legacy, or achieving personal fulfillment—their engagement is likely to be more enduring and intrinsically motivated (9, 10). Accordingly, this study incorporates meaning in life as a moderating variable to examine how existential meaning shapes the relationship between psychological processes and behavioral outcomes.

From a theoretical perspective, existing research on active aging rarely integrates spatial participation, psychological construction, and meaning-making within a single analytical framework. From a practical standpoint, the concept of co-construction and shared governance has become a key policy direction in China's urban development strategy (49). Against this backdrop, the present study seeks to bridge spatial regeneration and aging research by proposing a theoretically grounded and contextually relevant model.

In sum, this study investigates whether older adults' participation in the greening and regeneration of underutilized community spaces can promote active aging behavior through psychological mechanisms, namely landscape ownership and landscape emotional imprint. It further examines whether meaning in life moderates these relationships. By employing structural equation modeling, this research aims to elucidate the transformation mechanism linking participation, psychological processes, and behavior, Although the empirical setting is Chinese, the psychological mechanisms examined—landscape ownership, emotional imprint, and meaning in life—are not culturally specific, and the framework is intended to be transferable to other rapidly aging societies facing comparable challenges of community regeneration and active aging, thereby contributing to a deeper understanding of participatory spatial governance in aging societies.

## 2 Literature review and hypothesis development

### 2.1 Theoretical foundations

This study examines how older adults' participation in the greening and regeneration of underutilized community spaces is translated into active aging behavior through a set of psychological processes. In doing so, it develops an integrated framework that brings together four complementary theoretical perspectives: place attachment theory, self-determination theory, meaning-making in aging, and participatory design. Rather than treating these perspectives as independent lenses, this study positions them as jointly explaining how spatial participation becomes psychologically internalized and behaviorally enacted.

#### 2.1.1 Place attachment as the foundation of psychological landscape construction

Place attachment refers to the emotional, cognitive, and symbolic bonds that individuals develop with specific environments (11, 42) as well as a place-based attitudinal orientation encompassing affective, cognitive, and conative components (43). More recent work has expanded this tripartite person–process–place framework to incorporate dynamic and life-course perspectives, recognizing that attachment is continually renegotiated through everyday practices, social ties, and physical change in the environment (50, 51). For older adults in particular, this dynamic view is especially salient, as later-life transitions (e.g., retirement, bereavement, residential downsizing) reshape the meanings ascribed to familiar places. Prior research has emphasized that such attachment does not arise solely from the physical attributes of space, but is deeply rooted in lived experience and ongoing interaction with place. In other words, attachment is not simply a reaction to space—it is constructed through engagement with it. Building on this perspective, the present study conceptualizes landscape ownership and landscape emotional imprint as two key manifestations of place attachment in the context of community greening. When older adults participate in transforming underutilized spaces, they do not merely encounter an improved environment; rather, they inscribe personal meanings, memories, and emotional investments into that space. These processes foster a sense of belonging while simultaneously reinforcing one's perceived role within the community. As such, place attachment in this study is not treated as an endpoint, but as an evolving psychological condition that may shape subsequent behavioral engagement, particularly in the form of active aging.

#### 2.1.2 Self-determination theory and the internalization of participation

Self-Determination Theory (SDT) posits that human motivation is driven by the fulfillment of three basic psychological needs: autonomy, competence, and relatedness (12). When these needs are satisfied, externally initiated behaviors are more likely to be internalized and sustained over time. In the context of this study, participation in community greening serves as a critical interface through which older adults can experience autonomy and agency. When individuals perceive their involvement as self-directed rather than imposed, and when they are able to see the tangible outcomes of their contributions, participation becomes more than a task—it becomes meaningful action. This internalization process is central to understanding why some forms of participation lead to enduring behavioral change while others do not. Accordingly, this study positions perceived participation as the starting point of a broader psychological process. It is through this sense of involvement that individuals begin to develop emotional connections to space and a sense of ownership over it, ultimately translating into sustained and proactive forms of engagement.

#### 2.1.3 Meaning-making in aging as an existential driver of behavior

As individuals age, the search for meaning becomes increasingly salient. Meaning-making in aging highlights how older adults seek coherence, purpose, and continuity in their lives, often through reflection, contribution, and value realization (10). This perspective resonates with Tornstam's (13, 14) theory of gerotranscendence, which characterizes later life as a developmental shift from a materialistic and pragmatic worldview toward a more cosmic, transcendent, and relationally embedded mode of being—one in which meaning is increasingly derived from connectedness, contemplation, and the integration of past and present experience. Together, these perspectives suggest that meaning in later life is not merely a static psychological resource but an evolving existential orientation that shapes how older adults engage with their physical and social environments. Engagement in meaningful activities has been shown to enhance psychological integration and foster a stronger sense of existence and identity. Within this framework, participation in community space regeneration may acquire significance beyond its immediate functional outcomes. When older adults perceive their involvement as contributing to the community, leaving a legacy, or fulfilling personal values, their actions are more likely to be experienced as purposeful rather than incidental. For this reason, the present study introduces meaning in life as a moderating variable. It is expected that individuals with a stronger sense of meaning will be more likely to translate psychological connections with space into concrete behavioral expressions. In this sense, meaning does not simply amplify action; it shapes how psychological experiences are interpreted and enacted.

#### 2.1.4 Participatory design and the repositioning of older adults as co-creators

Participatory design emphasizes the active involvement of users in the creation and transformation of spaces, challenging the traditional model in which design decisions are made solely by professionals. Early work in this tradition demonstrated that grounding the design process in users' spatial reasoning—for example, through three-dimensional simulation models that allow non-experts to interrogate proposed schemes—substantially improves the legitimacy and usability of community design outcomes (15). Building on this foundation, contemporary scholarship reframes participation not as a discrete consultation event but as an ongoing socio-material practice. Bjögvinsson et al. (16) characterize participatory design as the cultivation of “design things”—evolving socio-material assemblies in which heterogeneous stakeholders, including non-traditional and previously marginalized user groups, continue to negotiate use and meaning long after the initial design moment. Dalsgaard (17) further argues that this orientation is especially pertinent in large-scale public projects, where institutional complexity, plural stakeholders, and long temporal horizons demand that participation be treated as a sustained infrastructural commitment rather than a one-off engagement exercise. By incorporating users' knowledge, experiences, and preferences in this sustained way, participatory processes can enhance both the relevance of design outcomes and users' sense of ownership and engagement (8). For older adults, participation in the regeneration of underutilized spaces represents more than physical involvement. It entails a reconfiguration of social roles—from passive recipients of services to active contributors to community development. Through participation, older adults are positioned not merely as users of space, but as co-creators of it. This perspective allows the present study to move beyond a stimulus–response understanding of environment and behavior. Instead of viewing space as a static context that influences health outcomes, this study conceptualizes space as something that is continuously shaped through participation, and in turn, shapes individuals' psychological states and behaviors. In doing so, it highlights the reciprocal relationship between spatial engagement and active aging.

### 2.2 Participation in green space regeneration

Participation in green space regeneration refers to the extent to which residents perceive themselves as active contributors in the transformation of underutilized community spaces. Rather than being limited to observable involvement, this construct captures a subjective experience encompassing perceived role, contribution, and meaning attached to one's participation. In this sense, participation is not merely behavioral—it reflects how individuals interpret their involvement within a broader social and spatial context. The conceptual roots of this construct can be traced to participatory design and place-making traditions, both of which emphasize the active role of users in shaping their environments. Within these perspectives, space is not treated as a finished product delivered to users, but as an evolving outcome co-created through ongoing interaction (8). In the context of community greening, participation may take the form of planning, planting, maintenance, or informal stewardship. Such involvement enables residents to move beyond passive consumption of space and develop a stronger sense of identification with it, which in turn fosters emotional attachment and sustained engagement (18).

At a deeper level, participation can also be understood through the lens of self-determination theory. When individuals perceive their involvement as voluntary, meaningful, and self-endorsed, participation is more likely to be internalized and sustained over time (12). This suggests that participation should not be conceptualized solely in terms of frequency or intensity, but also in terms of psychological significance. In particular, participation becomes consequential when it allows individuals to experience a sense of agency, competence, and social connection within their community environment. Building on these perspectives, participation in green space regeneration can be analytically understood along three interrelated dimensions. First, behavioral involvement reflects the extent to which individuals engage in concrete activities such as design, planting, or maintenance. Second, perceived recognition captures whether individuals feel that their contributions are acknowledged and valued by others. Third, perceived meaning refers to the extent to which participation is experienced as personally or socially meaningful. These dimensions align closely with the fulfillment of autonomy, competence, and relatedness needs, thereby providing a psychologically grounded framework for understanding participation among older adults.

Empirical evidence further supports the relevance of this construct in urban regeneration and aging contexts. For instance, Huo et al. (19) found that incorporating older residents' preferences into community green space design significantly increased both time spent in green areas and engagement in active behaviors. Similarly, Zhang and Xin (18) showed that resident-led micro-garden initiatives in Beijing not only improved environmental aesthetics but also strengthened neighborhood interaction and social cohesion. At the individual level, Lee (20), using a large sample of urban older adults in South Korea, demonstrated that involvement in green space use and improvement was associated with enhanced perceived health efficacy and life satisfaction.

Recent syntheses of participatory design practice across disciplines further consolidate this picture. Wacnik et al. (21), in a systematic review of participatory design research, identify recurring techniques—including co-design workshops, prototyping, scenario building, and stakeholder mapping—and emphasize that the methodological rigor and inclusivity of these techniques largely determine whether participation translates into substantive influence on design outcomes rather than tokenistic involvement. These insights are directly relevant to community green space regeneration, where the meaningfulness of older adults' participation depends not only on whether they are invited to participate, but on which techniques are deployed and how their contributions are integrated into design decisions. However, it is worth noting that a substantial portion of the existing literature continues to focus on green space exposure or usage frequency as primary predictors of wellbeing outcomes. Such approaches implicitly position residents as passive users of space, overlooking the role of participation in shaping both psychological attachment and behavioral sustainability. By foregrounding participation as a psychologically meaningful process, the present study seeks to extend this line of research and establish participation as a critical antecedent in the transformation from spatial engagement to active aging behavior.

### 2.3 Landscape ownership

Landscape ownership, also referred to as the psychological ownership of place, captures the extent to which individuals perceive a given environment as “mine” or “ours,” accompanied by a sense of control, responsibility, and personal investment. Unlike legal ownership, psychological ownership reflects a subjective state in which individuals develop a feeling of possession toward an object, organization, or space (22). In recent years, this concept has been extended to collective and spatial contexts, where it is increasingly used to explain how individuals relate to shared environments such as neighborhoods and public spaces. Although psychological ownership shares certain similarities with place attachment, the two constructs are conceptually distinct. Place attachment primarily emphasizes emotional bonding and symbolic meaning, whereas psychological ownership foregrounds perceived control, influence, and decision-making involvement. Empirical work in environmental psychology supports this distinction: Nijs et al. (23) show that collective psychological ownership of a neighborhood or shared park operates through two routes—a perceived right to determine outcomes, which can produce exclusionary tendencies, and a sense of group responsibility, which predicts stewardship behavior toward the place. In participatory contexts, the latter route is the substantively relevant one, as it captures the form of ownership that motivates individuals to care for and maintain shared spaces. In participatory contexts, the latter is particularly relevant, as it reflects a form of ownership that motivates individuals to care for and maintain shared spaces.

In this study, landscape ownership is conceptualized as a multidimensional construct comprising three interrelated components: a sense of entitlement or subjectivity, referring to the perception that one has a legitimate role in the space; perceived control, reflecting the extent to which individuals feel capable of influencing decisions or outcomes; and a sense of responsibility, indicating a willingness to care for and sustain the space. Together, these dimensions capture what may be described as spatial subjectivity—a psychological state in which individuals recognize themselves as active stakeholders in their environment. Importantly, this study positions landscape ownership not as a static attribute, but as an outcome of participatory processes. Drawing on participatory design theory, when individuals move from passive users to active contributors in shaping space, their relationship with that space undergoes a qualitative transformation (8). Participation enables individuals to see their ideas materialized, their efforts recognized, and their presence embedded in the environment. Over time, such experiences contribute to the emergence of a perceived “right to the space,” which is central to the development of psychological ownership.

This process can be further understood through psychological ownership theory. When individuals invest time, effort, and emotion into a target, and when they are able to exert some degree of control over it, the likelihood of developing ownership increases (22). In the context of community greening, participation provides precisely these conditions. Individuals who actively contribute to the transformation of underutilized spaces are more likely to perceive those spaces as extensions of themselves, rather than as externally provided environments. Empirical evidence supports this reasoning. Nijs et al. (24) found that residents who participated in the design and maintenance of urban green spaces reported significantly higher levels of psychological ownership and place-related loyalty than those who were merely users. Similarly, Huo et al. (19) demonstrated that participatory approaches in community greening projects among older adults enhanced not only their sense of responsibility but also their willingness to engage in ongoing maintenance activities—both of which are key indicators of ownership. Recent work further extends this line of inquiry to contested land-use settings, showing that psychological ownership shapes how individuals frame public-private boundaries in shared landscapes (41).

Taken together, these theoretical and empirical insights suggest that participation serves as a critical antecedent of landscape ownership. It is through engagement that individuals move from interaction with space to identification with it. Accordingly, when older adults actively participate in the regeneration of community green spaces, they are more likely to develop a stronger sense of ownership toward those spaces.

H1: Participation in green space regeneration is positively associated with landscape ownership.

### 2.4 Landscape emotional imprint

Landscape emotional imprint refers to the process through which individuals inscribe personal emotions, memories, and life experiences into a specific place, thereby forming enduring psychological traces and meaning structures associated with that environment. This concept draws on the intersection of affective bonding and place memory, emphasizing that emotional connections to space are not instantaneous but accumulate over time through lived experience, social interaction, and personal narrative (25). Unlike functional use or habitual exposure, emotional imprint reflects a deeper form of engagement in which space becomes intertwined with one's autobiographical memory and identity. Prior research suggests that such emotionally embedded landscapes can evoke multisensory recollections and reshape individuals' self-perception. For example, Monteiro et al. (26) found that older adults revisiting meaningful places experienced strong embodied memories that influenced both their spatial perception and identity reconstruction. Similarly, studies in Chinese urban contexts indicate that familiarity, memory activation, and sensory qualities of space significantly shape older adults' emotional states and wellbeing (27, 28).

In this study, landscape emotional imprint is conceptualized as comprising three interrelated dimensions: memory activation, referring to the extent to which a place evokes personal or social memories; emotional bonding, reflecting feelings of familiarity, comfort, and attachment; and identity projection, indicating the degree to which the place becomes part of one's personal narrative. Together, these dimensions form what may be described as a “spatial narrative system,” through which individuals interpret their relationship with the environment. For older adults in particular, such processes are closely linked to psychological continuity, identity maintenance, and emotional stability.

Building on place attachment theory, emotional connections to space are often rooted in active engagement rather than passive exposure (11). Participation provides opportunities for individuals to invest themselves in a place, interact with others, and accumulate meaningful experiences. In the context of community greening, older adults who engage in planning, planting, or maintaining shared spaces are more likely to develop emotional ties, as their personal efforts and social interactions become embedded in the environment. This process can also be understood through self-determination theory. When participation satisfies individuals' needs for autonomy and relatedness, it is more likely to be internalized as a meaningful experience rather than remaining a purely instrumental activity (12). As a result, participation becomes a vehicle through which emotions, memories, and self-relevant meanings are anchored in space. Empirical evidence supports this linkage. Monteiro et al. (26) demonstrated that involvement in public space design or regeneration can trigger autobiographical memory and identity-related reflection among older adults. Likewise, Zhang et al. (28) found that participation in community outdoor space management enhances emotional responses through sensory engagement, memory activation, and social interaction, thereby strengthening place attachment and wellbeing.

Taken together, these arguments suggest that participation in green space regeneration provides the experiential foundation for emotional imprint. Through active involvement, individuals move beyond mere interaction with space to the internalization of its emotional and symbolic significance.

H2: Participation in green space regeneration is positively associated with landscape emotional imprint.

Beyond its direct role, landscape emotional imprint may also serve as a precursor to the development of landscape ownership. While emotional attachment reflects a connection to place, ownership implies a stronger form of identification in which the place is perceived as part of the self. According to psychological ownership theory, individuals develop a sense of ownership when they invest time, effort, and identity into a target (22). Emotional imprint provides a critical pathway for such investment, as it anchors personal memories and meanings within the space. When a place becomes associated with one's life story, experiences, and values, it is more likely to be perceived as “mine” or “ours.”

Recent studies provide empirical support for this mechanism. Pierce et al. (22) identify investing the self into a target—through repeated use, personal effort, and accumulated experience—as one of the principal routes through which psychological ownership emerges, an account that maps directly onto the lived experience of community green space among long-tenured residents. Consistent with this, work on collective psychological ownership of neighborhoods and parks shows that prolonged engagement with a shared place fosters perceived group responsibility and willingness to maintain that place (23). In this sense, emotional imprint can be understood as a transitional process through which place attachment is transformed into psychological ownership. It is not sufficient for individuals to feel connected to a place; that connection must become internalized as part of their identity before ownership emerges. Accordingly, emotional imprint is expected to play a critical role in shaping landscape ownership.

H3: Landscape emotional imprint is positively associated with landscape ownership.

### 2.5 Active aging behavior

Active aging behavior refers to a set of sustained actions through which older adults maintain health, pursue personal fulfillment, and remain socially engaged across physical, psychological, and social domains. The concept originates from the World Health Organization's Active Aging Policy Framework, which reframes aging not as a process of decline, but as an ongoing process of participation, autonomy, and self-realization (1, 44). Within this perspective, older adults are viewed not as passive recipients of care, but as active agents capable of shaping their own wellbeing and contributing to society. Operationally, active aging encompasses a range of behaviors, including health maintenance, psychological adaptation, and social participation (9). These dimensions are commonly reflected in activities such as regular physical exercise, self-management of health, positive coping and self-development, as well as engagement in community and social networks. This multidimensional structure is consistent with frameworks such as the Active Aging Index (AAI), which conceptualize active aging as a behavioral and measurable construct rather than a purely abstract ideal.

A growing body of research highlights the broader societal relevance of active aging. For instance, Chang et al. (29), in a systematic review, noted that studies on active aging have increasingly focused on education, physical activity, and social participation across both European and East Asian contexts. In China, Chou et al. (30) demonstrated that participation in community-based learning groups significantly improved older adults' health behaviors, psychological attitudes, and social engagement. Similarly, Kalyoncuo and Kartin (31) found that healthy lifestyle practices—such as exercise, nutrition, and stress regulation—are strongly associated with active aging outcomes, reinforcing the view that active aging represents an integrated pattern of living rather than isolated behaviors. At the functional level, Park and Lee (32) further showed that higher active aging scores are closely linked to independence and daily functioning among older adults.

Despite these advances, much of the existing literature has focused on behavioral frequency or self-reported wellbeing as outcomes, with limited attention to the psychological mechanisms through which such behaviors are generated. In response, the present study conceptualizes active aging as the outcome of a broader psychological–spatial–behavioral process. Specifically, it examines how individuals' psychological relationships with space—formed through participation and emotional engagement—translate into sustained patterns of action. From this perspective, landscape ownership is expected to play a critical role in shaping active aging behavior. As a form of psychological ownership, it reflects a sense of control, responsibility, and personal investment in space (22). When individuals perceive a space as “theirs,” they are more likely to engage in behaviors that maintain, protect, and enhance that space. In community contexts, such ownership is closely linked to stewardship, participation, and proactive engagement (23, 33).

For older adults, the experience of ownership may be particularly consequential. Self-determination theory identifies autonomy (the experience of acting volitionally) and competence (the experience of effectance in one's environment) as basic psychological needs whose satisfaction sustains intrinsic motivation and long-term behavioral engagement (12, 34). Psychological ownership theory, in turn, proposes that perceived control over a target is one of the core routes through which individuals come to experience that target as part of the self (22). Bringing these two perspectives together, a perceived sense of control over one's community environment can be understood as a concrete, place-based source of the autonomy and competence experiences on which sustained engagement in health-related and social behaviors depends. Consistent with this reasoning, empirical work on older adults' environmental engagement reports that perceived agency and stewardship in shared spaces are associated with more frequent use of those spaces and with continued social participation (19, 35). When community spaces are experienced as extensions of the self, participation in those spaces is therefore likely to rely less on external incentives and more on these internalized motivational resources. Accordingly, landscape ownership is expected to be positively associated with a broad range of active aging behaviors, including health maintenance, social participation, and self-directed activity.

H4: Landscape ownership is positively associated with active aging behavior.

In addition to ownership, emotional imprint is also expected to contribute to active aging behavior through a distinct psychological pathway. Research on affective attachment suggests that emotionally meaningful environments are more likely to elicit sustained engagement and positive behavioral responses. When individuals associate a place with personal memories, social interactions, or a sense of familiarity, that place becomes a source of comfort, identity, and motivation. Empirical studies support this view. Ratcliffe and Korpela (36) found that autobiographical memory and self-relevant experiences in familiar environments significantly enhance perceived restorative qualities, mediated by place identity. Similarly, Zhang et al. (28) showed that older adults' emotional responses to familiar community green spaces—shaped by sensory experience, memory, and perceived safety—are associated with increased frequency of use and engagement in social activities. Ziggiatti Monteiro et al. (26) further demonstrated that emotionally embedded place memories can guide behavioral choices and participation patterns among older adults.

In this sense, emotional imprint provides a pathway through which space becomes meaningful, and meaning, in turn, motivates action. For older adults, when a place is not only used but emotionally lived in, it becomes a site where identity, memory, and daily practice converge. Such environments are more likely to support ongoing engagement and contribute to the realization of active aging.

H5: Landscape emotional imprint is positively associated with active aging behavior.

### 2.6 Mediating mechanisms: psychological pathways linking participation and behavior

While participation has been widely associated with positive behavioral outcomes among older adults, its effects are rarely direct. Instead, participation typically operates through a set of intervening psychological processes that shape how individuals interpret and internalize their experiences. In this study, it is argued that participation in green space regeneration influences active aging behavior through two distinct yet complementary mechanisms: landscape ownership and landscape emotional imprint. From the perspective of psychological ownership theory, participation provides individuals with opportunities to invest themselves in a target through time, effort, and influence (22). Such investment fosters a sense of ownership, which in turn promotes responsibility and sustained engagement. In the context of public green spaces, Noël and Dardenne (33) demonstrate that psychological ownership predicts both behavioral loyalty and proactive maintenance behaviors. Extending this logic to older adults, when individuals perceive themselves as legitimate stakeholders in a space, their actions are more likely to reflect long-term commitment rather than episodic involvement. In this sense, ownership functions as a mechanism through which participation is translated into stable and self-directed forms of behavior, including health maintenance, social engagement, and autonomous activity.

At the same time, participation may also operate through an affective pathway. Drawing on place attachment theory, emotional connections to space emerge through repeated interaction, memory formation, and personal experience (11). These emotional ties transform space into a meaningful context that supports identity and wellbeing. Empirical studies suggest that such processes are particularly salient among older adults. For instance, Monteiro et al. (26) and Zhang et al. (28) show that emotional memories associated with space can enhance both attachment and behavioral engagement. Within this framework, landscape emotional imprint represents a process through which individuals internalize the emotional significance of a place, thereby strengthening their motivation to remain engaged and active within it.

Taken together, these arguments suggest that participation does not automatically lead to active aging behavior. Rather, it must be psychologically translated into forms of ownership and emotional meaning before it can generate sustained action. Landscape ownership and emotional imprint thus serve as two key mediating pathways—one grounded in control and responsibility, the other in meaning and emotional connection. Both mechanisms enable individuals to move from external participation to internalized motivation. Accordingly, the following mediation hypotheses are proposed:

H6: Participation in green space regeneration is positively associated with active aging behavior through landscape ownership.

H7: Participation in green space regeneration is positively associated with active aging behavior through landscape emotional imprint.

### 2.7 Meaning in life as a moderating condition

Meaning in life refers to an individual's subjective sense that their existence is purposeful, valuable, and coherent. Within the framework of meaning-making theory, it is not merely an outcome of wellbeing but a fundamental psychological resource that shapes how individuals interpret experiences and sustain action over time (10, 37, 45). Particularly in later life, meaning becomes central to identity reconstruction, life evaluation, and the pursuit of continuity between past experiences and present engagement. In this study, meaning in life is conceptualized not as a direct predictor of behavior, but as a higher-order condition that determines whether psychological experiences are translated into sustained action. While both landscape ownership and emotional imprint represent powerful psychological connections to space, these connections do not necessarily lead to consistent behavioral outcomes. Instead, their effects depend on whether individuals are able to situate these experiences within a broader framework of personal meaning.

From the perspective of psychological ownership, a sense of “mine” or “ours” may foster responsibility and engagement (22). However, ownership alone does not guarantee enduring or value-driven behavior. When ownership is accompanied by meaning—such as perceiving one's involvement as a contribution to the community, a form of legacy, or an expression of personal worth—it becomes qualitatively different. In such cases, behavior is no longer driven by situational involvement but by internalized purpose. Empirical evidence supports this view. Pham and Bhar (38) show that internal psychological resources, including meaning in life, enhance individuals' capacity to translate cognitive and emotional states into adaptive and sustained behavior. Thus, meaning in life is expected to strengthen the relationship between landscape ownership and active aging behavior by transforming responsibility into purpose-driven action.

A similar argument applies to the affective pathway. Emotional imprint reflects the extent to which individuals integrate memories, emotions, and personal narratives into a specific place (36). While such emotional connections can foster attachment, they may remain passive unless anchored in a broader sense of meaning. For older adults, spaces that carry emotional significance—such as memories, relationships, or identity markers—can become sources of motivation only when these experiences are interpreted as meaningful components of one's life story. Without this interpretive layer, emotional attachment may lead to nostalgia or affective comfort, but not necessarily to sustained engagement or proactive behavior.

Meaning in life thus functions as a catalyst that converts psychological connection into behavioral continuity. It provides a framework through which both ownership and emotional attachment are elevated from situational experiences to value-oriented action. In this sense, meaning does not simply intensify existing relationships; it fundamentally alters their motivational quality. Accordingly, the following moderation hypotheses are proposed:

H8: Meaning in life positively moderates the relationship between landscape ownership and active aging behavior, such that the relationship is stronger at higher levels of meaning in life.

H9: Meaning in life positively moderates the relationship between landscape emotional imprint and active aging behavior, such that the relationship is stronger at higher levels of meaning in life.

**Figure 1:**
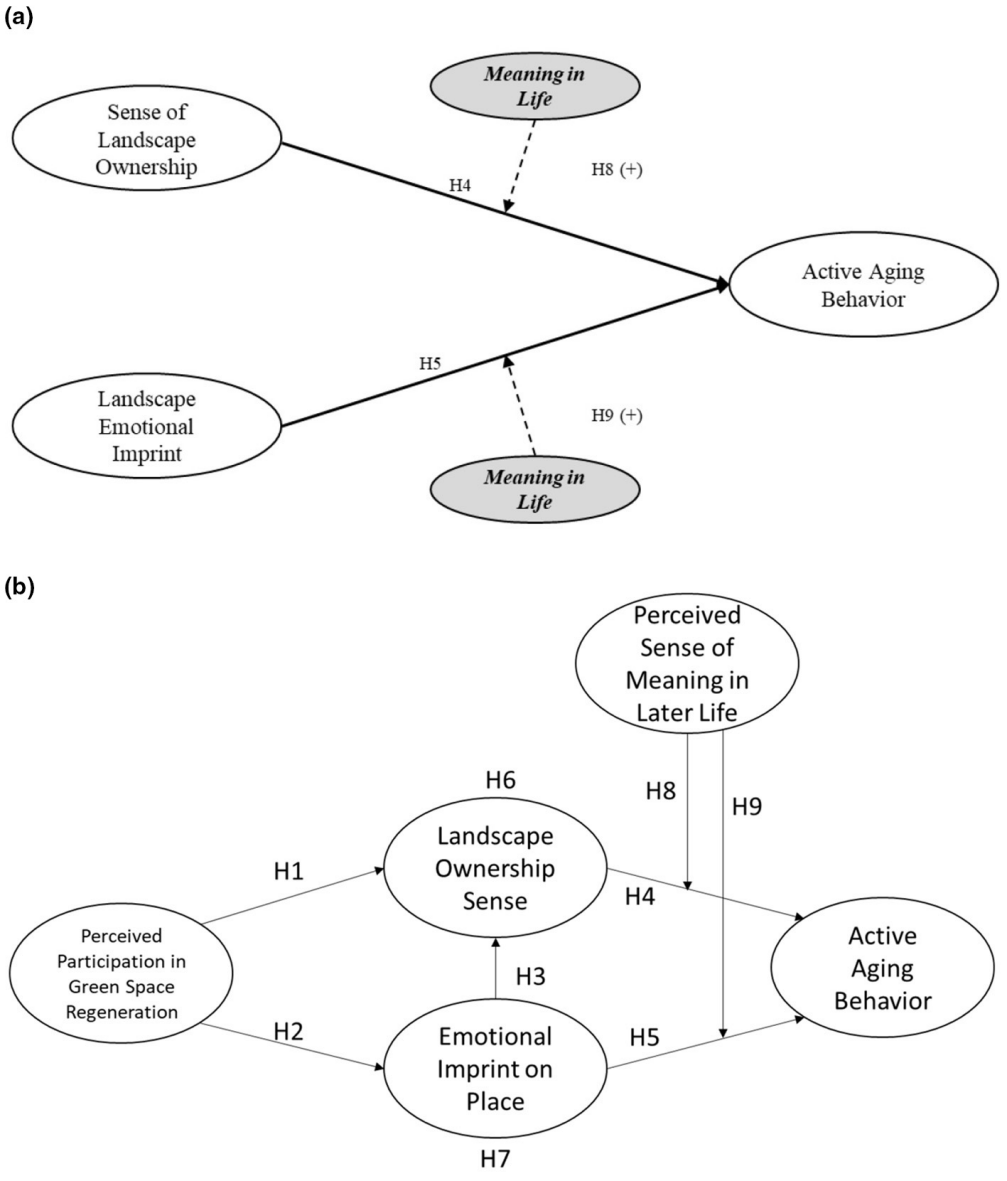
**(a)** Hypothesized moderating role of meaning in life on the psychological pathways to active aging behavior. **(b)** Hypothesized structural model.

The hypothesized moderating structure is summarized in Figure 1a, and the overall research framework is presented in Figure 1b.

## 3 Methodology

### 3.1 Participants and data collection

**Figure 2:**
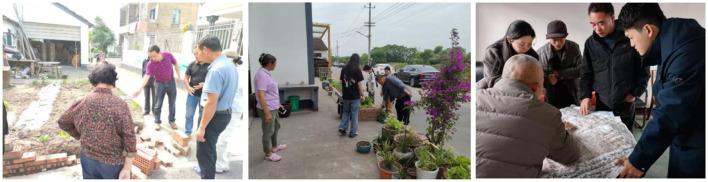
On-site collaborative greening practices in community space regeneration.

The target population of this study consisted of older adults residing in urban and semi-urban communities in China, particularly those living in neighborhoods where idle space regeneration or community greening initiatives had been implemented or were ongoing. To illustrate the empirical context of this study, field photographs were included to document community green space regeneration activities in China (see Figure 2). The images show older adults participating in the discussion, planning, and on-site implementation of idle space transformation. All photographs were taken by the research team during fieldwork. Written informed consent was obtained from individuals appearing in the photographs for the use and publication of identifiable images. All images were used in a manner that respects participants' privacy and dignity.

Participants were required to meet several inclusion criteria. Respondents had to be aged 60 or above, have resided in the community for at least one year, and possess sufficient cognitive and literacy abilities to complete the questionnaire. The age threshold of 60 years was chosen rather than 65 for two reasons. First, it is consistent with the statutory definition of older adults in the Law of the People's Republic of China on the Protection of the Rights and Interests of the Older Adults, which establishes 60 as the entry point of older adulthood and aligns with the official retirement-age framework. Second, this threshold is consistent with the great majority of empirical active-aging research conducted in China and other East Asian contexts [e.g., (5, 30)], thereby facilitating comparability with prior work. Participation was voluntary, and informed consent was obtained. A stratified sampling strategy was adopted to ensure representation across gender, education, and community type. Specifically, eight residential communities located in Ningbo and two adjacent prefecture-level cities in Zhejiang Province were purposively selected as primary sampling units on the basis of three criteria: (a) documented implementation of at least one community green space regeneration or micro-renewal initiative within the past three years; (b) heterogeneity in neighborhood typology, encompassing traditional danwei-style compounds, commodity-housing estates, and mixed-tenure neighborhoods, so as to capture variation in residents' prior exposure to participatory processes; and (c) accessibility through local neighborhood committees, who acted as liaison partners. Within each selected community, respondents were stratified by gender (male/female), educational attainment (three bands: primary or below, junior/senior secondary, tertiary), and length of residence ( ≤ 5 years, >5 years), and quotas were assigned proportionally based on community-level demographic records. Trained research assistants, accompanied by neighborhood-committee staff, recruited respondents on-site in shared community spaces (e.g., plazas, activity rooms) and through resident-organization introductions. A total of 512 older residents were invited to participate; of these, 467 agreed and completed the questionnaire, and 433 questionnaires were retained as valid after excluding 34 responses that were incomplete, exhibited straight-lining patterns, or failed the two embedded attention-check items, yielding an effective response rate of 84.6%. Data were collected using a structured questionnaire. Both self-administered and assisted formats were employed to accommodate differences in participants' literacy and physical conditions. Fieldwork was conducted on-site in collaboration with local community organizations, which helped improve response rates and data reliability. A total of approximately 433 valid responses were targeted to ensure sufficient statistical power. An a priori sample-size assessment was conducted using G^*^Power 3.1 for a linear multiple regression with five predictors, which corresponds to the maximum number of arrows pointing at any endogenous construct in the proposed model [the recommended rule of thumb for PLS-SEM sample-size planning; (52)]. Assuming a small-to-medium effect size (f^2^ = 0.10), α = 0.05, and statistical power of 0.95, the minimum required sample was 170. The obtained sample of 433 therefore exceeds the G^*^Power-based minimum by more than 2-fold and also clears the more conservative “10 cases per predictor” threshold (52), indicating adequate statistical power for estimating the structural model, mediation paths, and interaction terms.

### 3.2 Measurement instruments

#### 3.2.1 Demographic variables

To provide a comprehensive profile of the sample and to support subsequent statistical analyses, a set of demographic variables was included in the questionnaire. These variables were selected not only for descriptive purposes but also for their established relevance in aging research, particularly in relation to health behavior, social participation, and environmental engagement. Specifically, seven demographic characteristics were measured: gender, age, educational attainment, marital status, living arrangement, length of residence in the community, and self-rated health. All variables were operationalized using closed-ended categorical formats to ensure clarity for respondents and consistency in data analysis. Gender was assessed with inclusive response options to respect diversity and personal preference. Age was grouped into five-year intervals beginning at 60 (i.e., 60–64, 65–69, 70–74, 75–79, 80 and above), allowing for the identification of potential differences across stages of later life. Educational attainment was included as an indicator of socio-cultural resources, which may influence individuals' cognitive engagement and participation patterns. Marital status and living arrangement were incorporated to capture variations in social support and daily life structure, both of which have been shown to play critical roles in older adults' wellbeing and activity levels.

Length of residence was measured as an indicator of familiarity with the local environment and accumulated place-based experience, which is particularly relevant in studies involving spatial perception and community participation. In addition, self-rated health was assessed using a five-point scale ranging from very poor to very good. This measure has been widely recognized as a reliable proxy for overall health status and a strong predictor of behavioral outcomes in aging populations. Together, these demographic variables were used to describe the sample characteristics, examine potential group differences, and serve as control variables in subsequent analyses. Their inclusion enhances both the internal validity of the study and the interpretability of the findings across different subgroups.

#### 3.2.2 Construct measurement

The study examined five key constructs: perceived participation in green space regeneration, landscape ownership, landscape emotional imprint, meaning in life, and active aging behavior. All constructs were measured using a seven-point Likert scale ranging from 1 (strongly disagree) to 7 (strongly agree). This scaling approach is widely adopted in behavioral and social science research due to its sensitivity in capturing variations in subjective perceptions, attitudes, and behavioral tendencies. It is also particularly suitable for structural equation modeling, as it allows for robust estimation of latent constructs. All variables were modeled as reflective constructs, with multiple items designed to capture different facets of the same underlying concept. The measurement development followed established procedures, including theoretical grounding, adaptation from prior studies where applicable, and contextual refinement to fit the specific setting of community-based green space regeneration among older adults. For each construct, an initial item pool was generated either by adapting validated scales from the prior literature (meaning in life, landscape ownership, active aging behavior) or, for context-specific constructs without established instruments (perceived participation in green space regeneration, landscape emotional imprint), by developing new items grounded in the conceptual dimensions identified in Section 2. The initial item pool was first subjected to content validity review by a five-member expert panel comprising two professors in environmental design, one professor in gerontology, one senior community planner, and one community-based social worker with extensive fieldwork experience among older adults. Experts independently rated each item for clarity, relevance, and conceptual fit on a four-point scale, following the content validity index (CVI) procedure recommended by Polit and Beck (39); items with an item-level CVI below 0.80 were revised or removed. All surviving items were then back-translated between English and Simplified Chinese by two bilingual researchers to ensure semantic equivalence. A pilot test was subsequently conducted with 32 older adults drawn from two communities that were not included in the main survey. The pilot aimed to evaluate item comprehension, response burden, and scale reliability in the target population. Cronbach's α for each construct in the pilot exceeded 0.80, and cognitive-interview feedback from ten pilot respondents led to minor wording simplifications (e.g., replacing abstract terms with concrete examples familiar to older residents) before the finalized instrument was administered in the main study.

##### 3.2.2.1 Perceived participation in green space regeneration

Perceived participation was operationalized as older adults' subjective evaluation of their involvement in the planning, design, decision-making, and implementation of community green space regeneration (see Section 2.2 for the conceptual development of this construct, building on the theoretical foundations in Section 2.1). Five items were developed for this study, capturing perceived involvement, opportunity for expression, actual engagement, perceived contribution, and participation in decision-making. Items were drafted by the research team and refined through expert review and pilot testing as described above.

##### 3.2.2.2 Landscape ownership

Landscape ownership was operationalized as older adults' perceived psychological ownership over community green spaces—a symbolic sense of “mine” or “ours” encompassing identification, control, participation rights, and felt responsibility, rather than legal title (see Section 2.3 for theoretical grounding in psychological ownership theory and its integration with place attachment). Five items were adapted from Pierce et al. (22) and contextualized for the community green space setting, capturing sense of control, personal investment, identification with the space, perceived participation rights, and responsibility for maintenance.

##### 3.2.2.3 Landscape emotional imprint

Landscape emotional imprint was operationalized as the extent to which a specific place has become integrated with individuals' emotions, memories, and life experiences (see Section 2.4 for the theoretical positioning within place-attachment and memory-based literatures). Five items were developed for this study to capture memory evocation, emotional connection, narrative identification, sense of familiarity, and perceived personal meaning associated with the place.

##### 3.2.2.4 Meaning in life

Meaning in life was operationalized as older adults' perceived sense that their current activities, roles, and experiences remain purposeful, valuable, and coherent (see Section 2.1.3 for the theoretical grounding in meaning-making perspectives, and Section 2.7 for its moderating role in the present model). Five items were adapted from the presence-of-meaning subscale of the Meaning in Life Questionnaire [MLQ; (10)], with wording refined to align with the context of community participation and environmental engagement.The measurement focuses on the presence-of-meaning subscale, consistent with prior research in aging contexts.

##### 3.2.2.5 Active aging behavior

Active aging behavior was operationalized at the behavioral level as the extent to which older adults engage in sustained actions supporting their physical health, psychological wellbeing, and social participation (see Section 2.5 for the theoretical grounding in the WHO active aging framework and related empirical literature). Five items were adapted from the Active Aging Index framework (9) and contextualized for community settings, covering three dimensions: (1) health-related behaviors (physical activity, personal health management); (2) psychological and self-development behaviors (positive outlook, purposeful activity); and (3) social participation behaviors (interaction with others, involvement in community activities).

### 3.3 Data analysis

This study employed partial least squares structural equation modeling (PLS-SEM) using SmartPLS 4.0 to analyze both the measurement and structural models. PLS-SEM was considered appropriate for this research for several reasons. First, the study aims to examine a complex model involving multiple latent constructs, including mediation and moderation effects. Second, several constructs in this study are conceptually integrative and context-specific, making PLS-SEM suitable due to its flexibility in handling prediction-oriented models and theory development (52). Third, PLS-SEM is robust for relatively moderate sample sizes and does not require strict assumptions of multivariate normality. The analysis proceeded in two stages. In the first stage, descriptive statistics were conducted to summarize the sample characteristics. Subsequently, the measurement model was assessed to evaluate the reliability and validity of the constructs. Indicator reliability was examined through outer loadings, with values above 0.70 considered acceptable. Internal consistency reliability was assessed using composite reliability (CR), while convergent validity was evaluated based on the average variance extracted (AVE), with a threshold of 0.50. Discriminant validity was examined using both the Fornell–Larcker criterion and the heterotrait–monotrait (HTMT) ratio. Given that the constructs in this study were theoretically grounded and adapted from established literature, exploratory factor analysis (EFA) was not conducted. Instead, the focus was placed on confirmatory assessment within the PLS framework. In the second stage, the structural model was evaluated to test the proposed hypotheses. Bootstrapping with 5,000 resamples was performed to assess the significance of path coefficients. The coefficient of determination (R^2^) was used to evaluate the explanatory power of the model, while effect sizes (f^2^) were calculated to assess the relative impact of each predictor. In addition, mediation effects were examined through indirect effect analysis, and moderation effects were tested using interaction terms within the PLS framework.

Given that all variables in this study were measured using self-reported data collected at a single time point, common method bias (CMB) was a potential concern (53). Both procedural and statistical remedies were implemented to mitigate this risk. Procedurally, respondents were assured of the anonymity and confidentiality of their responses, informed that there were no “right” or “wrong” answers, and presented with predictor and criterion items in separated sections of the questionnaire to reduce consistency motifs and demand characteristics. Item wording was kept concise and concrete to minimize ambiguity. Statistically, two complementary diagnostics were performed. First, Harman's single-factor test was conducted by entering all 25 measurement items into an unrotated principal-components analysis. The first extracted factor accounted for 62.36% of the total variance, exceeding the conventional 50% threshold. We note, however, that Harman's test has been widely criticized as overly sensitive and insufficient on its own, particularly when latent constructs are theoretically expected to covary strongly—as is the case in the present sequential mediation model linking participation, ownership, emotional imprint, meaning, and active aging behavior (40, 53). We therefore relied principally on the more rigorous full collinearity assessment proposed by Kock (54), in which variance inflation factors (VIFs) are computed for each latent construct against all others within the PLS framework. The resulting VIFs were 2.324 (PGS), 2.494 (SLO), 2.837 (EIP), 2.564 (SML), and 2.088 (AAB)—all comfortably below the conservative threshold of 3.3. Taken together with the procedural remedies described above, the VIF results indicate that common method bias is unlikely to pose a substantive threat to the validity of the findings, although the limitations of single-source, single-time-point self-report data are acknowledged in Section 5.4.

## 4 Results

### 4.1 Sample characteristics

A total of 433 valid responses were collected for analysis. The demographic profile of the sample is summarized in Table 1. In terms of gender distribution, female respondents (56.8%) slightly outnumbered male respondents (43.2%). Regarding age, the majority of participants were between 60 and 69 years old (66.3%), followed by those aged 70–79 (32.8%), while respondents aged 80 and above accounted for less than 1% of the sample. This indicates that the sample is primarily composed of young-old and middle-old adults. With respect to educational attainment, most respondents reported relatively low levels of formal education. Specifically, 37.9% had completed primary school and 43.0% had completed junior high school, accounting for over 80% of the sample. Only a small proportion held college degrees (11.1%) or postgraduate qualifications (1.4%).

**Table 1:** Sample demographic characteristics (*N* = 433).

Category	Group	Frequency	Percentage (%)
Gender	Male	187	43.2
	Female	246	56.8
Age	60–69	287	66.3
	70–79	142	32.8
	80 and above	4	0.9
Education level	No formal education	28	6.5
	Primary school	164	37.9
	Junior high school	186	43.0
	High school/vocational school	1	0.2
	College/university	48	11.1
	Postgraduate and above	6	1.4
Marital status	Married	428	98.8
	Single	1	0.2
	Other	4	0.9
Living arrangement	Living alone	3	0.7
	Living with spouse	211	48.7
	Living with family (multiple members)	209	48.3
	Other	10	2.3
Length of residence	Less than 1 year	36	8.3
	1–5 years	146	33.7
	6–10 years	246	56.8
	10–20 years	5	1.2

In terms of marital status, the sample was highly homogeneous, with 98.8% of respondents being married. Similarly, living arrangements were largely family-oriented, with most participants living either with a spouse (48.7%) or with family members (48.3%), while only a small fraction lived alone (0.7%). Finally, regarding length of residence, the majority of respondents had lived in their communities for 6–10 years (56.8%), followed by those with 1–5 years of residence (33.7%). This suggests that most participants had sufficient familiarity with their local environments to meaningfully evaluate community space and participation experiences.

### 4.2 Measurement model

#### 4.2.1 Convergent validity

The results of the measurement model assessment are presented in Table 2. Convergent validity was evaluated by examining outer loadings, internal consistency reliability, and the average variance extracted (AVE). All indicator loadings ranged from 0.864 to 0.975, exceeding the recommended threshold of 0.70, indicating satisfactory indicator reliability (52). In addition, Cronbach's alpha values ranged from 0.950 to 0.978, and composite reliability (CR) values ranged from 0.962 to 0.983, both well above the recommended cutoff of 0.70, demonstrating a high level of internal consistency (52, 55). Furthermore, the AVE values for all constructs ranged from 0.834 to 0.919, exceeding the minimum requirement of 0.50 (56). These results collectively confirm that the measurement model exhibits strong convergent validity.

**Table 2:** Convergent validity of measurement model.

Construct	Item	Outer loading	Cronbach's α	CR	AVE
Active aging behavior	AAB1	0.953	0.953	0.964	0.843
	AAB2	0.921			
	AAB3	0.919			
	AAB4	0.888			
	AAB5	0.909			
Emotional imprint on place	EIP1	0.970	0.978	0.983	0.919
	EIP2	0.959			
	EIP3	0.975			
	EIP4	0.968			
	EIP5	0.921			
Perceived participation in green space regeneration	PGS1	0.912	0.954	0.965	0.845
	PGS2	0.931			
	PGS3	0.927			
	PGS4	0.912			
	PGS5	0.914			
Sense of landscape ownership	SLO1	0.891	0.950	0.962	0.834
	SLO2	0.953			
	SLO3	0.864			
	SLO4	0.903			
	SLO5	0.951			
Meaning in life	MIL1	0.934	0.965	0.973	0.878
	MIL2	0.942			
	MIL3	0.941			
	MIL4	0.963			
	MIL5	0.904			

#### 4.2.2 Discriminant validity

Discriminant validity was assessed using the Fornell–Larcker criterion, as presented in Table 3. The results indicate that the square root of the average variance extracted (AVE) for each construct (diagonal elements) exceeds its correlations with other constructs. This satisfies the criterion proposed by Fornell and Larcker (56), suggesting that each construct is empirically distinct from the others. In addition, the inter-construct correlations were all below critical thresholds, indicating no concerns regarding multicollinearity or construct overlap. These findings provide further support for the discriminant validity of the measurement model (52).

**Table 3:** Discriminant validity (Fornell–Larcker Criterion).

Construct	AAB	EIP	PGS	MIL	SLO
Active aging behavior (AAB)	**0.918**				
Emotional imprint on place (EIP)	0.677	**0.959**			
Perceived participation (PGS)	0.583	0.647	**0.919**		
Meaning in life (MIL)	0.634	0.727	0.641	**0.937**	
Sense of landscape ownership (SLO)	0.602	0.671	0.708	0.654	**0.913**

Bold values on the diagonal are the square root of the average variance extracted (AVE) for each construct.

### 4.3 Model evaluation

The explanatory power of the structural model was evaluated using R^2^ values, following the guidelines of (52). The results indicate that the model demonstrates moderate to substantial explanatory power. Specifically, perceived participation in green space regeneration explains 41.9% of the variance in emotional imprint on place (R^2^ = 0.419), while emotional imprint on place and perceived participation jointly explain 58.0% of the variance in sense of landscape ownership (R^2^ = 0.580). Furthermore, emotional imprint on place and sense of landscape ownership together account for 55.4% of the variance in active aging behavior (R^2^ = 0.554). These findings suggest that the proposed model possesses strong predictive capability and satisfactory explanatory power.

### 4.4 Structural model assessment

**Figure 3:**
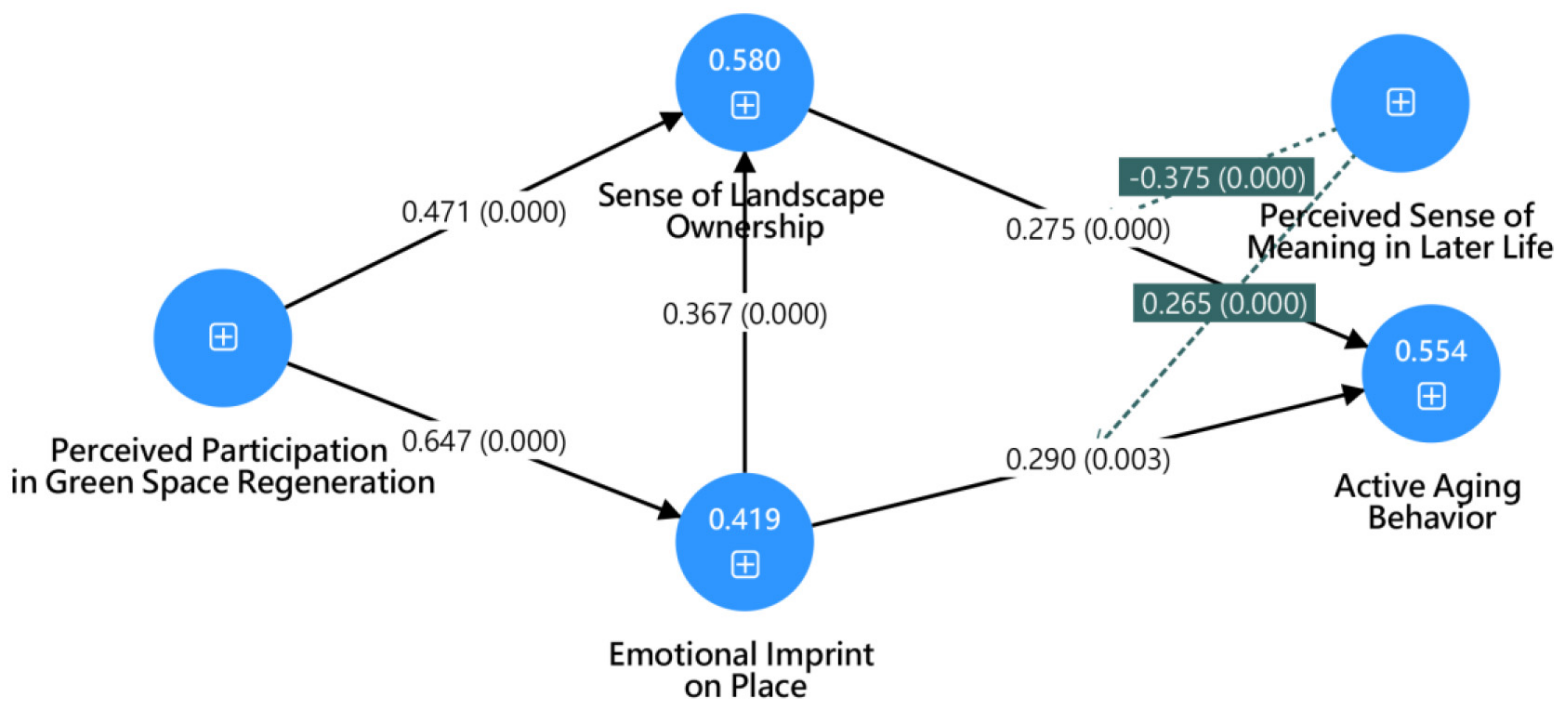
PLS-SEM statistical model diagram.

The structural model was assessed using partial least squares structural equation modeling (PLS-SEM), which is well suited for examining complex relationships and prediction-oriented research frameworks (52, 57). The results indicate that perceived participation in green space regeneration exerts a strong and statistically significant positive effect on emotional imprint on place (β = 0.647, t = 18.699, *p* < 0.001). This relationship explains 41.9% of the variance in emotional imprint on place (R^2^ = 0.419), indicating a moderate level of explanatory power. Emotional imprint on place further demonstrates a significant positive influence on sense of landscape ownership (β = 0.367, t = 4.831, *p* < 0.001). At the same time, perceived participation in green space regeneration shows an additional direct effect on sense of landscape ownership (β = 0.471, t = 8.180, *p* < 0.001). Together, these variables account for 58.0% of the variance in sense of landscape ownership (R^2^ = 0.580), suggesting substantial explanatory power. With respect to behavioral outcomes, both emotional imprint on place (β = 0.290, t = 2.947, *p* = 0.003) and sense of landscape ownership (β = 0.275, t = 4.527, *p* < 0.001) significantly predict active aging behavior. The model explains 55.4% of the variance in active aging behavior (R^2^ = 0.554), indicating strong predictive capability, as shown in Table 4 and Figure 3.

**Table 4:** Structural model results.

Path relationship	Original sample	Standard deviation	t statistics	*P* values	R2
Perceived Participation in Green Space Regeneration → Emotional Imprint on Place	0.647	0.035	18.699	0.000	0.419
Emotional Imprint on Place → Sense of Landscape Ownership	0.367	0.076	4.831	0.000	0.580
Perceived Participation in Green Space Regeneration → Sense of Landscape Ownership	0.471	0.058	8.180	0.000	
Emotional Imprint on Place → Active Aging Behavior	0.290	0.098	2.947	0.003	0.554
Sense of Landscape Ownership → Active Aging Behavior	0.275	0.061	4.527	0.000	

### 4.5 Mediation analysis

The mediating effects were examined using the bootstrapping procedure recommended for PLS-SEM (52, 58). The results reveal that emotional imprint on place serves as a significant mediator in the relationship between perceived participation in green space regeneration and active aging behavior (β = 0.188, t = 2.879, *p* = 0.004). The 95% bias-corrected confidence interval does not include zero [0.051, 0.308], supporting the presence of a statistically significant indirect effect. Similarly, sense of landscape ownership was found to significantly mediate the relationship between perceived participation in green space regeneration and active aging behavior (β = 0.129, t = 3.726, *p* < 0.001), with a corresponding confidence interval of [0.061, 0.197]. These results suggest that both affective attachment to place and psychological ownership mechanisms play meaningful roles in linking participation experiences to behavioral outcomes, as shown in Table 5.

**Table 5:** Mediation effects.

Mediation effects	Original sample	Standard deviation	*T* statistics	*P* values	2.50%	97.50%
Perceived Participation in Green Space Regeneration → Emotional Imprint on Place → Active Aging Behavior	0.188	0.065	2.879	0.004	0.051	0.308
Perceived Participation in Green Space Regeneration → Sense of Landscape Ownership → Active Aging Behavior	0.129	0.035	3.726	0.000	0.061	0.197

### 4.6 Moderation analysis

**Figure 4:**
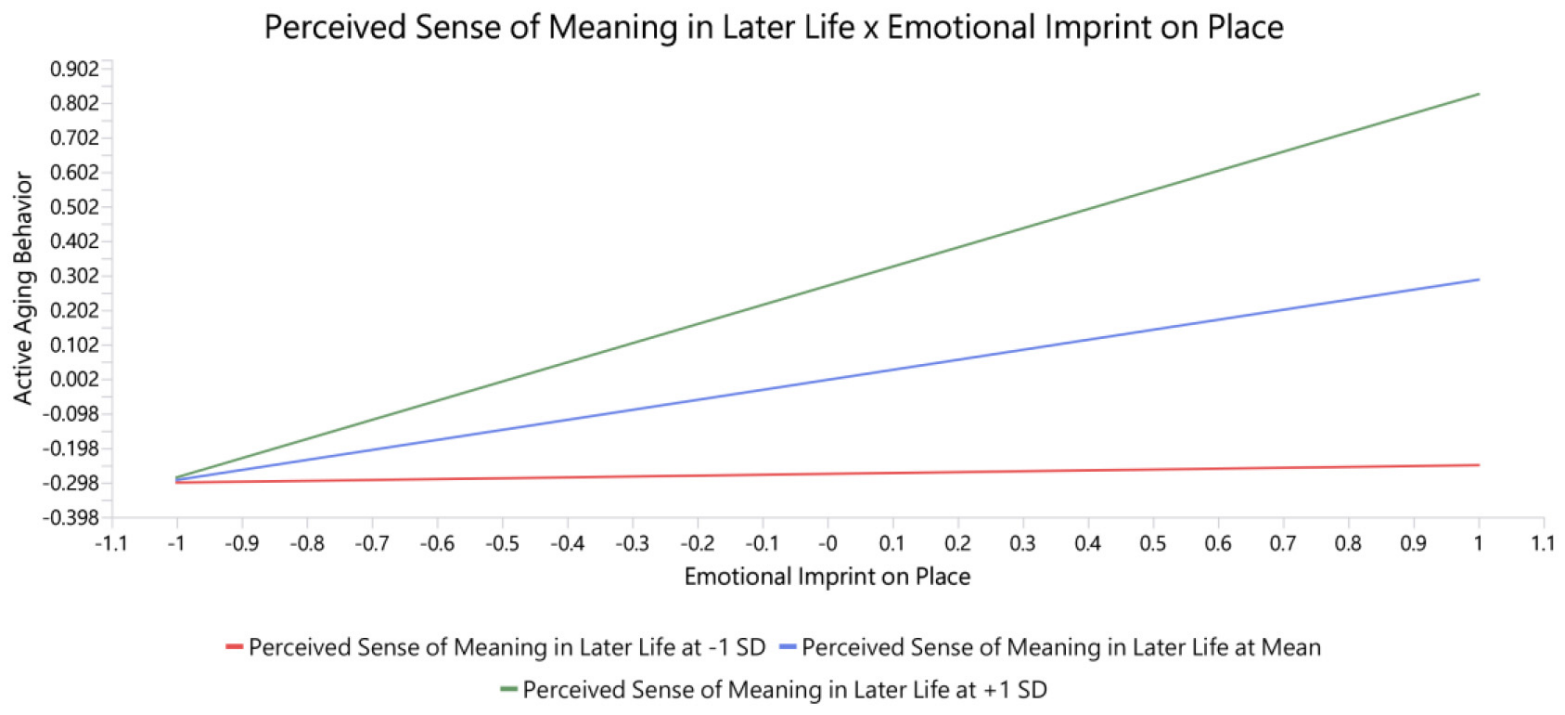
Perceived sense of meaning in later life x emotional imprint on place → active aging behavior.

**Figure 5:**
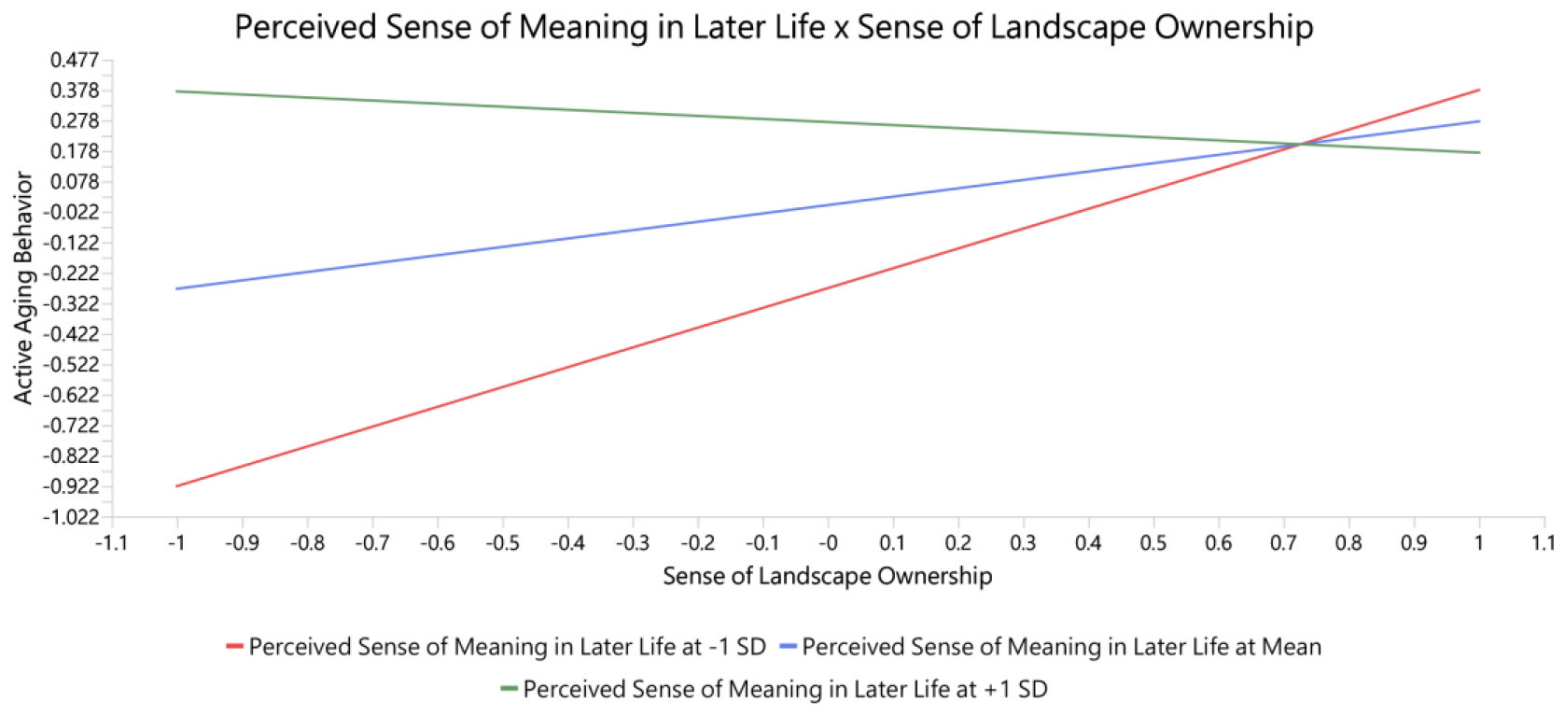
Perceived sense of meaning in later life x sense of landscape ownership → active aging behavior.

The moderating effects were examined using the product indicator approach with bootstrapping, following established procedures for moderation analysis in PLS-SEM (52, 59). The results indicate that perceived sense of meaning in later life significantly strengthens the relationship between emotional imprint on place and active aging behavior (β = 0.265, t = 3.511, *p* < 0.001). This suggests that when individuals perceive a stronger sense of meaning in their later life, the positive influence of emotional attachment to place on active aging behavior becomes more pronounced. In other words, emotional experiences associated with place are more likely to be translated into sustained behavioral engagement when they are embedded within a broader sense of life meaning. In contrast, perceived sense of meaning in later life exhibits a significant negative moderating effect on the relationship between sense of landscape ownership and active aging behavior (β = −0.375, t = 4.509, *p* < 0.001), as shown in Table 6, Figures 4, 5.

**Table 6:** Moderation effects.

Moderation effects	Original sample	Standard deviation	*T* statistics	*P* values
Perceived Sense of Meaning in Later Life x Emotional Imprint on Place → Active Aging Behavior	0.265	0.075	3.511	0.000
Perceived Sense of Meaning in Later Life x Sense of Landscape Ownership → Active Aging Behavior	−0.375	0.083	4.509	0.000

With respect to the hypothesized moderation effects, the results indicate that H9 (meaning in life positively moderates the emotional imprint → active aging behavior path) was supported, whereas H8 (meaning in life positively moderates the landscape ownership → active aging behavior path) was not supported in the hypothesized direction; instead, the moderation was significant but negative. This pattern, although contrary to the original prediction, is theoretically interpretable. When older adults already derive a strong sense of meaning from their later life, the additional motivational contribution of ownership-based responsibility appears to attenuate rather than amplify, suggesting a substitution rather than an amplification mechanism: meaning in life and landscape ownership may function as alternative, partially redundant motivational resources for sustaining active aging behavior. Older adults with high levels of meaning in life are likely to be intrinsically motivated and may rely less on ownership-derived obligation, whereas those with lower levels of meaning may draw more strongly on landscape ownership as a compensatory source of purpose. The contrasting pattern across the two moderated paths (positive amplification for the affective pathway, negative substitution for the ownership pathway) is further interpreted in Section 5.1, and graphically depicted in Figures 4, 5; the implications for differentiated intervention design are developed in Section 5.4.

Figure 4 plots the simple slopes of emotional imprint on active aging behavior at low (one standard deviation below the mean) and high (one standard deviation above the mean) levels of meaning in life. Both slopes are positive, consistent with H5, but the two lines diverge as emotional imprint increases: the slope is visibly steeper at high meaning in life than at low meaning in life, and the gap between the two lines widens at the right-hand end of the x-axis. This fan-shaped pattern indicates an amplification form of moderation—older adults who already derive a strong sense of meaning from later life convert affective attachment to community green spaces into sustained active-aging behavior more readily, whereas for those with weaker meaning the same level of emotional imprint is translated into behavior to a lesser degree. Substantively, the positive interaction (β = 0.265, *p* < 0.001) suggests that emotional connection to place is not self-activating: it reliably supports sustained behavioral engagement primarily when the individual can situate those affective experiences within a broader narrative of personal purpose.

Figure 5 plots the analogous simple slopes for landscape ownership. In contrast to Figure 4, the two lines diverge in opposite directions: at low meaning in life the slope of active aging behavior on ownership is clearly positive, whereas at high meaning in life the slope is markedly shallower and, at the upper range of ownership, the two lines cross. This crossover pattern is the visual signature of the negative interaction estimated in the structural model (β = – 0.375, *p* < 0.001), and it supports a substitution rather than an amplification interpretation of the moderation. For older adults with lower meaning in life, landscape ownership functions as a compensatory motivational resource—a sense of “mine” or “ours” that supplies responsibility and direction when an internal sense of purpose is less available. For older adults with higher meaning in life, intrinsic purpose already motivates sustained behavior, so the incremental motivational contribution of ownership-based responsibility is smaller and, at very high levels of ownership, the two resources appear partially redundant. The contrast between Figures 4, 5 thus indicates that meaning in life does not uniformly strengthen all psychological pathways to active aging: it amplifies the affective (emotional imprint) pathway while substituting for the cognitive-agentic (ownership) pathway.

## 5 Discussion

### 5.1 Key findings and integrated mechanism

This study examines how older adults' participation in community green space regeneration is associated with active aging behavior through underlying psychological mechanisms. Because the data are cross-sectional, the pathways reported below should be interpreted as patterns of association consistent with the hypothesized mechanism rather than as established causal effects. Within this constraint, the findings are consistent with an integrated pathway in which participation is not directly associated with behavioral outcomes; rather, its association with behavior appears to operate through a process of psychological internalization involving emotional imprint on place and sense of landscape ownership. Specifically, perceived participation is positively associated with both emotional imprint on place and sense of landscape ownership, suggesting that engagement in regeneration activities co-occurs with more meaningful psychological connections to the environment. Participation, therefore, should not be viewed merely as behavioral involvement, but as a process of meaning construction through which physical space becomes psychologically significant. Furthermore, emotional imprint on place positively influences sense of landscape ownership, suggesting a sequential relationship in which affective experiences serve as the foundation for identity-based connections to place.

Both emotional imprint on place and sense of landscape ownership are positively associated with active aging behavior, and the indirect effects through these two constructs are statistically significant, consistent with their role as mediating mechanisms. These results suggest that the association between participation and behavior appears to operate indirectly, through internal psychological processes, rather than through a direct behavioral route. Interpreted within the limits of the cross-sectional design, this pattern is consistent with a process in which participation co-occurs with a transition from external engagement to more intrinsically motivated actionand with sustained involvement in active aging practices. In addition, the moderating role of perceived meaning in life reveals a more nuanced motivational structure. Meaning in life strengthens the relationship between emotional imprint on place and active aging behavior, while weakening the relationship between sense of landscape ownership and behavior. This dual effect suggests that behavioral motivation in later life operates through multiple psychological pathways. When meaning in life is more salient, affective connections are more readily translated into action, whereas reliance on ownership-based mechanisms becomes less central.

### 5.2 Psychological mechanism of place construction

The findings of this study reveal that older adults' participation in community green space regeneration functions as a critical mechanism for psychological place construction, through which external environments are gradually internalized into meaningful components of the self. Rather than being a simple form of social or functional engagement, participation initiates a continuous psychological process that links affective experience, identity formation, and behavioral activation. From the perspective of self-determination theory (SDT), participation provides a context in which older adults can experience autonomy and competence through involvement in decision-making and hands-on activities. Such experiences foster intrinsic motivation, allowing individuals to engage with their environment not out of obligation, but through self-endorsed and meaningful action. This process contributes to the formation of emotional imprint on place, as repeated interactions and personal investment generate affective bonds with the environment. Building on place attachment theory, these affective bonds emerge through ongoing interaction and lived experience within the space. Activities such as planting, maintenance, and collaborative space-making enable older adults to accumulate personal memories and embed their life experiences into the environment. Through this process, space is transformed from a functional setting into a psychologically meaningful place, where emotional attachment reflects not only present engagement but also the integration of personal narratives over time.

Importantly, the findings further suggest that emotional imprint on place serves as a precursor to the development of sense of landscape ownership. Unlike conventional perspectives that emphasize control or possession as the basis of ownership, the present study highlights an affective pathway in which ownership emerges from accumulated emotional experience. As individuals develop deeper emotional connections with place, the boundary between self and environment becomes increasingly blurred, giving rise to a sense of identification in which the place is perceived as part of the self. In this sense, landscape ownership among older adults is less about control and more about experiential integration and identity extension. These psychological processes ultimately translate into behavioral outcomes. Emotional imprint on place represents an affective pathway, where behavior appears closely tied to emotional attachment and personal significance, while sense of landscape ownership reflects an identity-based pathway, grounded in responsibility and self-extension. Together, these pathways are consistent with a sequential and complementary structure linking participation to sustained active aging behavior.

Taken together, this study conceptualizes place construction as a dynamic psychological process in which participation is associated with the emergence of emotional connection, emotional connection co-develops with identity-based ownership, and these internalized relationships with place are linked to behavioral engagement. This integrated mechanism provides a more comprehensive understanding of how everyday environmental interaction contributes to active aging in later life.

### 5.3 Theoretical contributions

This study makes several important theoretical contributions by integrating spatial participation with psychological mechanisms to advance understanding of active aging, place attachment, and psychological ownership. First, this study extends the active aging literature by introducing a place-based psychological perspective. While prior research has predominantly explained active aging through health conditions, social support, or institutional arrangements, the present study finds that participation is not directly associated with behavioral outcomes. Instead, its association with behavior appears to operate through internal psychological processes, specifically emotional imprint on place and sense of landscape ownership. By examining how participation is linked to sustained action through psychological internalization, this study addresses a key gap in understanding the mechanisms underlying active aging behavior. Second, this study advances place attachment theory by proposing a sequential psychological process linking affective connection to identity formation. Existing research has often treated emotional attachment and place identity as parallel constructs; however, the present findings suggest that emotional imprint on place functions as a precursor to the development of landscape ownership. This sequential perspective provides a more dynamic understanding of how place-based meanings evolve from affective experience to identity integration.

Third, this study refines psychological ownership theory by highlighting the role of affective experience in later-life contexts. Traditional perspectives emphasize control, self-investment, and knowledge as primary antecedents of ownership. In contrast, the present study shows that among older adults, ownership emerges primarily from accumulated emotional experiences and lived engagement with place. This finding suggests that psychological ownership is context-dependent and underscores the importance of affective pathways in shaping ownership perceptions in aging populations. This position both extends and contrasts with recent work. Whereas Noël and Dardenne (33) demonstrate that psychological ownership predicts loyalty toward public green spaces in a general adult sample, and Nijs et al. (23) locate the behavioral force of collective ownership in a sense of group responsibility that motivates stewardship of neighborhoods and parks, the present findings suggest that in later-life contexts the pathway is more specifically affective-experiential: emotional imprint functions as a precursor to ownership rather than as a parallel construct. Relative to Hill et al.'s (8) qualitative account of older adults' participation in urban regeneration—which foregrounds the relational and recognition-seeking dimensions of participation—the present study supplies a complementary quantitative mechanism that links those relational experiences to sustained active-aging behavior through identifiable psychological mediators. Taken together, these comparisons reinforce the argument that the psychological correlates of ownership and attachment are not uniform across populations, and that aging populations merit theorization in their own right. The sequential emotional-imprint-to-ownership pathway is consistent with Scannell and Gifford's (11) tripartite place-attachment framework in treating affective bonds as foundational to identity-based ties with place, but specifies a temporal ordering that the tripartite model leaves underdetermined. By embedding these strands in a single internalization mechanism and by testing them jointly in a structural model, the present study offers an integrative account rather than a competing one. Finally, this study proposes an integrated theoretical framework linking participation, psychological place construction, and behavior. By conceptualizing a “participation–internalization–behavior” mechanism, the study moves beyond direct-effect models and demonstrates that behavioral outcomes are shaped through sequential and complementary psychological processes. Furthermore, the identification of meaning in life as a higher-order moderating mechanism highlights the dynamic nature of motivation in later life, showing how different psychological pathways are differentially activated depending on individuals' existential orientation. Taken together, this study develops a psychologically grounded and integrative framework that connects place, emotion, identity, and meaning, offering a more comprehensive explanation of active aging behavior and opening new avenues for future research on aging and place-based experience.

### 5.4 Practical implications

The findings of this study provide several practical implications for community planning, urban regeneration, and aging-related policy design. First, community regeneration initiatives should shift from infrastructure-oriented approaches toward participatory design. The results show that older adults' involvement in green space regeneration not only improves environmental conditions but also facilitates psychological place construction. Therefore, practitioners should create opportunities for meaningful participation, enabling older adults to act as co-creators rather than passive users of community spaces. Second, emotional engagement should be incorporated into spatial design and program planning. Given the critical role of emotional imprint on place in sustaining behavioral engagement, community interventions should emphasize activities that foster affective connections, such as incorporating local memories, shared experiences, and intergenerational interaction. These approaches help transform functional spaces into psychologically meaningful environments that support long-term participation. This is consistent with recent evidence from China showing that social participation moderates the relationship between urban green space and the mental health of older adults (46).

**Figure 6:**
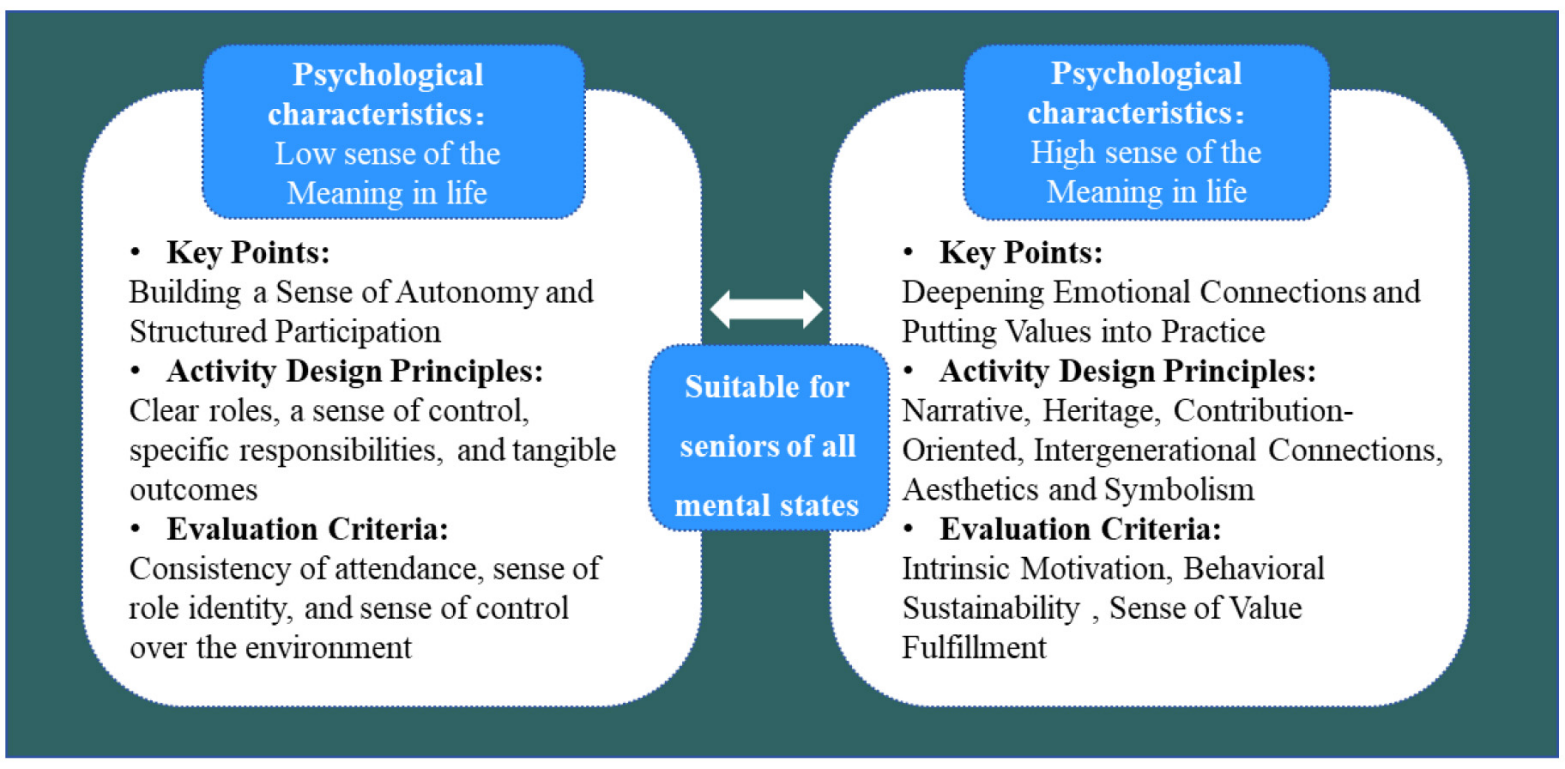
An analysis of differentiation strategies.

Third, differentiated intervention strategies are necessary to account for variations in meaning in life among older adults. As illustrated in Figure 6, two complementary pathways can be identified. For individuals with lower levels of meaning in life, structured participation, clear roles, and tangible outcomes are particularly effective in strengthening their sense of control and role identity. In contrast, for those with higher levels of meaning in life, interventions that emphasize emotional connection, value realization, and purpose-driven engagement are more effective in promoting sustained behavioral involvement.

Importantly, Figure 6 highlights that these strategies should not be interpreted as rigid categories but as part of a dynamic continuum. Older adults may shift between different motivational states over time, suggesting that flexible and adaptive intervention design is essential. Overall, the findings indicate that effective age-friendly community development should move beyond the provision of physical space toward fostering psychological connection, enabling older adults to transition from passive presence to active contribution.

### 5.5 Limitations and future research

Despite its theoretical and empirical contributions, this study has several limitations that should be acknowledged. First, the cross-sectional design limits the ability to establish causal relationships among participation, psychological place construction, and active aging behavior. Future research is encouraged to adopt longitudinal or experimental designs to examine the dynamic evolution of these relationships over time. Second, although both procedural remedies (anonymity assurances and separation of predictor and criterion items in the questionnaire) and statistical diagnostics (Harman's single-factor test and full collinearity VIF assessment) were employed to mitigate common method bias, the use of self-reported data collected at a single time point may not entirely eliminate this concern. Future studies could incorporate multiple data sources, such as behavioral observations or third-party assessments, to enhance robustness. Third, the sample is context-specific, focusing on older adults in selected communities in China, which may limit the generalizability of the findings. Cross-cultural and multi-regional studies are therefore needed to validate the applicability of the proposed framework in diverse contexts. Fourth, the sample skewed heavily toward the young-old and middle-old categories (99.1% aged 60–79), with only four respondents (0.9%) aged 80 or above. While this age distribution is broadly consistent with the population of community-dwelling older adults actively engaged in outdoor community affairs in urban China, it constrains the generalizability of the findings to the old-old population. Physical-mobility limitations, cognitive changes, and narrower social networks among the oldest-old may shape participation opportunities and the psychological internalization of participation in qualitatively different ways; the ownership pathway and emotional imprint pathway identified here may therefore operate with different relative salience in that subgroup. Purposive sampling of the old-old, and designs that accommodate alternative modes of participation such as observation, narrative recollection, or intergenerational co-engagement, are accordingly needed to extend the framework to the full spectrum of later life.

Building on these limitations, several directions for future research are proposed. Future studies may further explore how different types of spatial environments (e.g., parks, community gardens, cultural spaces) influence psychological place construction and behavioral outcomes. In addition, incorporating individual-level factors such as health status, social networks, and socioeconomic conditions may provide a more comprehensive understanding of behavioral motivation in later life. Qualitative approaches, including in-depth interviews and narrative analysis, are also recommended to capture the nuanced processes through which older adults construct meaning and emotional connections with place.

## 6 Conclusion

In conclusion, this study demonstrates that older adults' participation in community green space regeneration does not directly translate into active aging behavior, but operates through a process of psychological internalization involving emotional imprint on place and sense of landscape ownership. Furthermore, meaning in life plays a critical moderating role in shaping how these psychological pathways influence behavior. By conceptualizing a “participation–internalization–behavior” mechanism, this study highlights that active aging is not merely driven by external engagement, but by the extent to which individuals develop meaningful connections with their environment. In this sense, space should be understood not only as a physical setting, but as a psychological medium through which older adults construct identity, sustain motivation, and enhance their quality of life.

## Data Availability

The datasets generated and analyzed during the current study are publicly available in the Zenodo repository at https://doi.org/10.5281/zenodo.20140050.
